# Suppression of the transforming growth factor-β signaling pathway produces a synergistic effect of combination therapy with programmed death receptor 1 blockade and radiofrequency ablation against hepatic carcinoma in mice

**DOI:** 10.1080/21655979.2022.2051688

**Published:** 2022-03-30

**Authors:** Xiaoxiang Fan, Lihu Gu, Shuyi Lv, Meiwu Zhang, Luhui Zhuang, Yan Zhang, Ping Chen

**Affiliations:** a Department of Interventional Therapy, HwaMei Hospital, University of Chinese Academy of Sciences, Ningbo, Zhejiang, China; b Ningbo Institute of Life and Health Industry, University of Chinese Academy of Sciences, Ningbo, Zhejiang, China; c Key Laboratory of Diagnosis and Treatment of Digestive System Tumors of Zhejiang Province, Ningbo, Zhejiang, China; d Department of General Surgery, HwaMei Hospital, University of Chinese Academy of Sciences, Ningbo, Zhejiang, China

**Keywords:** Primary liver cancer, radiofrequency ablation, PD-1, TGF-β

## Abstract

Primary liver cancer (PLC) significantly affects the health of patients globally owing to its high morbidity and low survival rate. Radiofrequency ablation (RFA) has recently been introduced for the clinical treatment of PLC. However, significant immunosuppressive effects are induced by RFA, which limits its application. This study aimed to explore the potential of combination therapy with RFA by investigating the effects of siRNAs against programmed death receptor 1 (PD-1) and transforming growth factor-β (TGF-β) on the antitumor effect induced by RFA. We observed that compared with si-NC, cell viability was reduced, apoptosis rate was elevated, release of inflammatory factors and percentage of CD3^+^CD8^+^ cells were increased, and the PI3K/AKT/mTOR pathway was repressed in the co-culture of RFA-treated H22 cells and CD8^+^ T cells by transfection with si-PD-1 and si-TGF-β; these effects were further enhanced by co-transfection with si-PD-1 and si-TGF-β. Additionally, in H22 cell xenograft-bearing mice treated with RFA, compared with the si-NC group, repressed tumor growth, prolonged survival, increased production of inflammatory factors and expression of CD3 and CD8 in tumor tissues, and downregulation of the PI3K/AKT/mTOR pathway were observed in the si-PD-1 and si-TGF-β groups; these effects were further enhanced in the si-PD-1 + si-TGF-β group. Taken together, our data revealed that suppression of the TGF-β signaling pathway produced a synergistic antitumor effect of combination therapy with PD-1 blockade and RFA against PLC.

**Figure:**
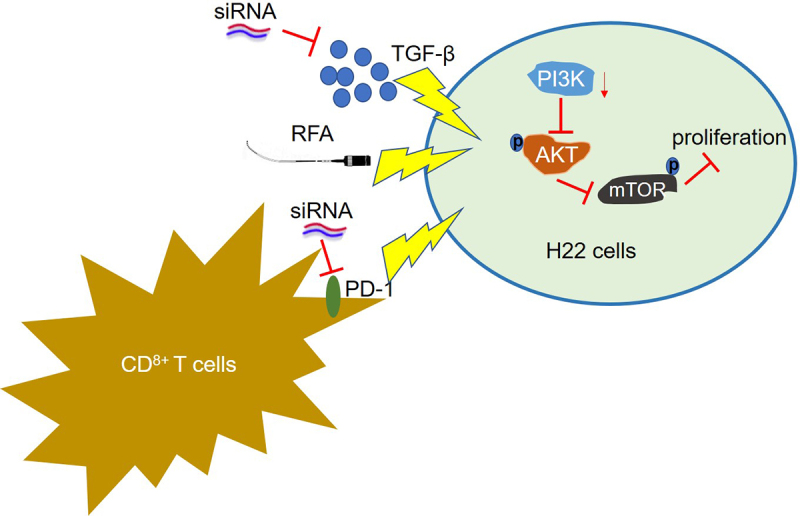

## Introduction

Primary liver cancer (PLC) is a common malignant tumor with increased morbidity in recent years [1]. Currently, the clinical treatments for PLC include surgery, radiofrequency ablation, hepatic arterial chemoembolization, radiotherapy, immune checkpoints [2,3], and targeted drug therapy [4]; among these alternatives, radical surgical excision has been the first-line treatment. However, 80% of patients with PLC are first diagnosed at either intermediate or advanced phase, and most patients have complications such as cirrhosis and abnormal liver function, which are not tolerant to radical surgical excision [5]. Radiofrequency ablation (RFA) is a minimally invasive *in situ* tumor treatment technology. Guided electrode needles are directly inserted into the tumor with the help of ultrasound or computed tomography imaging technology, and radiofrequency energy is used to generate high temperature that shrinks and destroys the soft tissue and tumor [6]. Compared to surgical resection, RFA has the advantages of being minimally invasive, having a little impact on liver function, simple, safe, and strong repeatability. RFA is gradually attracting attention as multidisciplinary comprehensive treatment method for PLC and provides a novel treatment method for patients who are not tolerant to radical surgical excision [7]. The comprehensive treatment method with RFA as the main treatment and in combination with various methods has gradually become the first-line choice for the treatment of PLC. Significant progress has been made in recent years. However, it is difficult to break through the bottleneck of high metastasis and recurrence rates after PLC treatment [8]. Therefore, more effective combination treatments are urgently needed.

With the development of immunotherapy, patients with PLC have significantly benefited from immune checkpoint blockade therapy represented by programmed cell death protein 1 (PD-1) antibodies. However, the overall efficacy of this therapy is limited and is only observed in patients with high expression of programmed cell death ligand 1 (PD-L1) [9]. According to an in-depth study of the PLC tumor microenvironment, tumor cells evade immune monitoring *via* several mechanisms and interact with immune cells and inflammatory factors in the liver to produce a unique immunosuppressive microenvironment inside the tumor [10]. Myeloid-derived suppressor cells (MDSCs) are proliferated and activated after the development of liver progenitor cell, which further secrete interleukin 10 (IL-10) and activate regulatory T (Treg) cells. This results in increased expression of immune checkpoints that repress the immune response of T cells [11]. PD-L1 is upregulated in tumor cells *via* activation of the transforming growth factor beta (TGF-β) signaling pathway, the inactivation of which contributes to improved efficacy of PD-1 antibodies. TGF-β is a multifunctional polypeptide cytokine that regulates cell growth, mediates cell phenotype, and inhibits tumor growth *in vivo* and *in vitro* [12].

The present study was aimed to prove the hypothesis that inactivation of the TGF-β signaling pathway in the tumor microenvironment exerts a positive synergistic effect on PD-1 antibody treatment combined with RFA against PLC. The present study elucidates the mechanistic role of TGF-β, PD-1, and RFA against tumor, and provides basis for the development of more effective combination therapies for the clinical treatment of PLC to bring benefits to patients.

## Materials and methods

### Cells and animals

H22 cells (mouse PLC cell line) and CD8^+^ T cells were purchased from Novo Biotechnology Co., Ltd. (Beijing, China) and cultured in Dulbecco’s modified Eagle’s medium supplemented with 10% fetal bovine serum under 5% CO_2_ and 37°C. Thirty male C57BL/6 mice were obtained from Beijing Vital River Laboratory Animal Technology Co., Ltd. (Beijing, China).

### RFA treatment

When the cell density reached 1 × 10^7^ cells/well and the tumor size reached 100 mm^3^, RFA was performed using EPT-1000 XPTM cardiac radiofrequency generator (Boston Scientific, Massachusetts, USA), equipped with a 4 mm cardiac ablation probe, according to a previously described method [13]. The performance test was conducted under defined conditions: a power output of 80°C for a duration of 60s.

### Transfection[14]

To achieve PD-1 knockdown and TGF-β knockdown in H22 cells, the cells were transfected with siRNA targeting mouse PD-1 (si-PD-1) and siRNA targeting mouse TGF-β (si-TGF-β) for 48 h, respectively. Lipofectamine 3000 (Invitrogen, Carlsbad, CA, USA) was used as the transfection reagent, and si-NC was used as the negative control. The sequences for si-PD-1 and si-TGF-β were 5′-CCAGGAUGGUUCUUAGACUUU-3′ and 5′- CCGCAACAACGCCATCTATGA-3′, respectively.

### In vitro *assay*

When the cell density reached 1 × 10^7^ cells/well, H22 cells were co-cultured with 1 × 10^6^ mouse CD8^+^ T cells and subjected to RFA in the presence or absence of si-PD-1, si-TGF-β, or si-NC for 24 h, followed by collection of cells for subsequent functional assays.

### MTT assay[15]

Cells were incubated with 0.25 mg/mL MTT solution (R&D Systems, Minneapolis, MN, USA) at 37°C for 3 h and the culture medium was removed, followed by the addition of dimethyl sulfoxide to produce blue formazan. Finally, absorbance at 630 nm was measured using a microplate reader (Mindray, Shenzhen, China).

### Flow cytometry[16]

Apoptosis rate of cells was evaluated by flow cytometry. The cells were collected and detected using FITC Annexin V Apoptosis Detection Kit I (BD Pharmingen, San Jose, CA, USA) according to the manufacturer’s instructions. Finally, the cells were loaded into BD flow cytometer (BD Pharmingen) after filtration for apoptosis analysis.

The percentage of CD3^+^CD8^+^ cells was measured by flow cytometry. In brief, cells were collected and resuspended in phosphate-buffered saline, followed by the addition of anti-CD3 antibodies (1:1000; R&D Systems) for 30 min in the dark at 4°C. After washing thrice, the cells were incubated with anti-CD8 antibodies (1:1000; R&D Systems) for 30 min in the dark at 4°C. After washing thrice, the cells were transferred to a flow tube and loaded into BD flow cytometer (BD Pharmingen) to determine the percentage of CD3^+^CD8^+^ cells.

### ELISA[17]

ELISA was used to measure the levels of IL-6, IL-1β, tumor necrosis factor alpha (TNF-α), and TGF-β in the cell supernatant. Briefly, test samples and standards were seeded in 96-well plates, followed by incubation with the conjugate reagents for 1.5 h. After adding 3,3ʹ5,5ʹ-tetramethylbenzidine solution to each well, the samples were incubated at 37°C for 15 min. Finally, the stop solution was added to terminate the reaction, and absorbance was measured at 450 nm using a microplate reader (Mindray).

### Western blot analysis[18]

After isolating total proteins from cells or tumor tissues, proteins were quantified using the bicinchoninic acid kit (Ziker, Guangdong, China), followed by loading into 12% SDS-PAGE. After separating for 1.5 h, proteins were transferred to a polyvinylidene fluoride membrane (Invitrogen, CA, USA), followed by incubation in tris-buffered saline with Tween 20 (TBST) buffer containing 5% bovine serum albumin (BSA). Then, the primary antibodies against PD-1 (1:1000; R&D Systems), PD-L1 (1:1000; R&D Systems), PI3K (1:1000, R&D Systems), AKT (1:1000; R&D Systems), p-AKT (1:1000; R&D Systems), mTOR (1:1000; R&D Systems), p-mTOR (1:1000; R&D Systems), and glyceraldehyde 3-phosphate dehydrogenase (GAPDH) (1:1000; R&D Systems) were introduced, followed by the addition of the secondary antibody (1:2000; R&D Systems). Finally, the enhanced chemiluminescence reagent was added, and the bands were visualized using ImageJ software.

### Xenograft model[19]

H22 cells treated with si-NC, si-PD-1, or si-TGF-β were transplanted into C57BL/6 mice; when the tumor size reached 100 mm^3^, RFA was performed. The length (L), width (W), and weight of tumor tissues were measured at the end of animal experiments. The tumor volume (V) was calculated using the following formula: V = L × W^2^ × 0.5.

### Immunohistochemical analysis[20]

Briefly, tumor tissues were fixed with 4% paraformaldehyde, dehydrated, and embedded in paraffin. Subsequently, the tissues were cut into 5-μm sections, deparaffinized, and rehydrated. After incubation in 5% BSA for 30 min, the sections were incubated with primary antibodies against PD-1 (1:200; Abcam, Cambridge, UK), PD-L1 (1:200; Abcam), CD3 (1:200; Abcam), or CD8 (1:200; Abcam), followed by incubation with HRP-conjugated secondary antibody. Finally, images were captured using a light microscope (Laird Technologies, Inc., Missouri, USA). Five high-power fields were selected randomly for imaging of each slide.

### Statistical analysis

The data were analyzed using GraphPad software and expressed as the mean ± SD. Student’s *t*-test was used to compare data between two groups, whereas data among more than three groups were analyzed using one-way analysis of variance. Differences were considered statistically significant at p < 0.05.

## Results

We hypothesized that inactivation of the TGF-β signaling pathway in the tumor microenvironment exerts a positive synergistic effect on PD-1 antibody treatment combined with RFA against PLC. The present study aimed to explore a potential combination therapy having synergistic effect against PLC. We first explored the synergistic antitumor effect of co-treatment with RFA, si-PD-1, and si-TGF-β in H22 cells by determining the cell viability, apoptosis rate, cell proliferation, and cytotoxicity of CD^8+^ T cells and the activity of the PI3K/AKT/mTOR pathway. Subsequently, the synergistic antitumor effect of co-treatment with RFA, si-PD-1, and si-TGF-β was confirmed in a xenograft model by measuring the tumor growth, survival rate, infiltration of CD^8+^ T cells, and activity of the PI3K/AKT/mTOR pathway.

### Synergistic inhibitory effect against proliferation of H22 cells was observed after co-treatment with RFA, si-PD-1, and si-TGF-β

**Figure 1.**
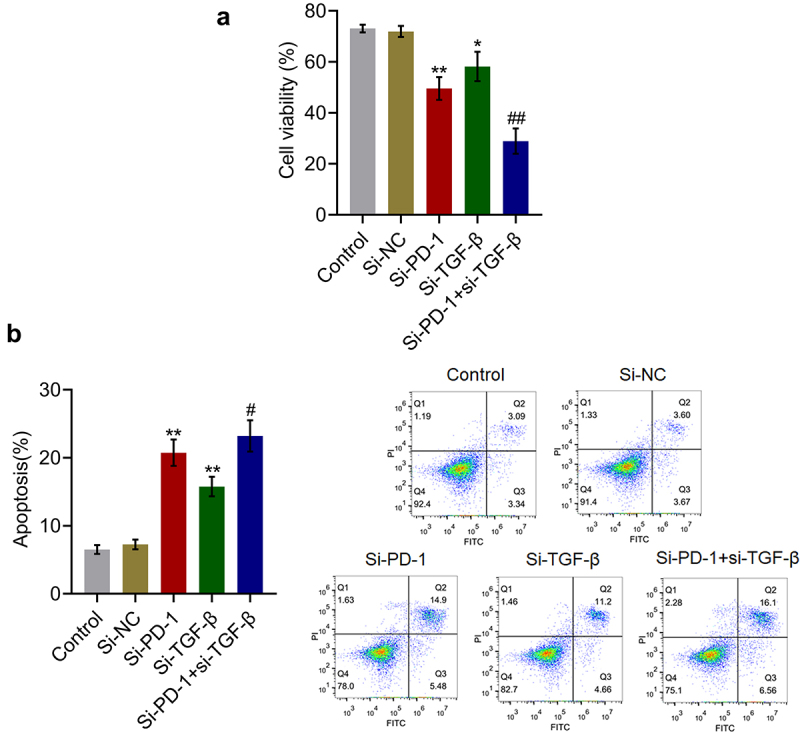
Proliferation of H22 cells was synergistically inhibited by si-PD-1 combined with si-TGF-β. RFA was performed on the co-culture of H22 and CD8^+^ T cells, followed by transfection with si-NC, si-PD-1, si-TGF-β, and si-PD-1 + si-TGF-β. A. The proliferation of the cells was evaluated by the MTT assay. B. The apoptosis rate was measured by flow cytometry (*p < 0.05 *vs*. si-NC, **p < 0.01 *vs*. si-NC, #p < 0.05 *vs*. si-PD-1, ##p < 0.01 *vs*. si-PD-1).

First, for *in vitro* assay, RFA of H22 and CD8^+^ T cells was performed in the presence of blank medium, si-NC, si-PD-1, si-TGF-β, or both si-PD-1 and si-TGF-β, and cell viability and apoptosis rate were determined. As shown in Figure 1a, compared with the si-NC group, cell viability was significantly reduced in the si-PD-1 and si-TGF-β groups. Moreover, compared to the si-PD-1 group, significantly reduced cell viability was observed in the si-PD-1 + si-TGF-β group (*p < 0.05 *vs*. si-NC, **p < 0.01 *vs*. si-NC, ##p < 0.01 *vs*. si-PD-1). In addition, compared with the control, the apoptosis rate (Figure 1b) was significantly elevated from 7.27% to 20.38% and 15.86% after transfection with si-PD-1 and si-TGF-β, respectively. Compared with the si-PD-1 group, the apoptosis rate was dramatically increased to 22.66% after co-transfection with si-PD-1 and si-TGF-β (**p < 0.*01 vs*. si-NC, #p < 0.05 *vs*. si-PD-1). These data indicated that the inhibitory effect of RFA on proliferation of PLC cells was synergistically enhanced by knockdown of PD-1 or TGF-β, particularly by knockdown of both PD-1 and TGF-β.

### TGF-β and PD-1/PD-L1 were suppressed by si-PD-1 and si-TGF-β

**Figure 2.**
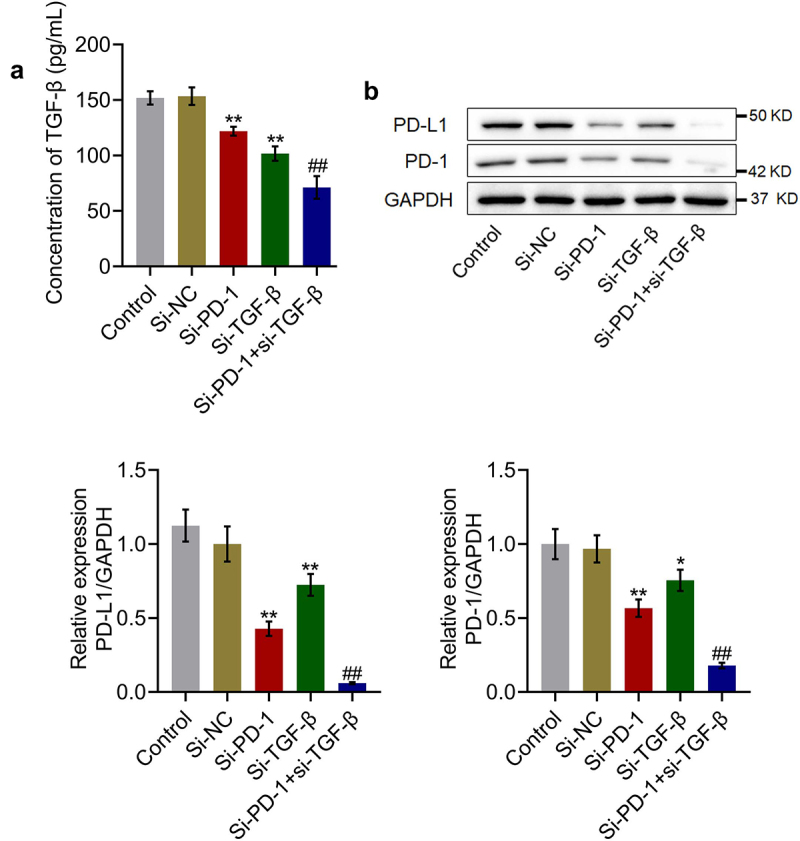
TGF-β and PD-1/PD-L1 expression was suppressed by si-PD-1 and si-TGF-β. RFA was performed on the co-culture of H22 and CD8^+^ T cells, followed by transfection with si-NC, si-PD-1, si-TGF-β, and si-PD-1 + si-TGF-β. A. The secretion of TGF-β was measured by ELISA. B. The expression levels of PD-1 and PD-L1 were determined by western blotting (*p < 0.05 *vs*. si-NC, **p < 0.01 *vs*. si-NC, ##p < 0.01 *vs*. si-PD-1).

To check the efficacy of knockdown of PD-1 and TGF-β, the expression levels of TGF-β and PD-1/PD-L1 were determined in the culture. As shown in Figure 2a, compared with the si-NC group, the production of TGF-β was significantly reduced from 153.5 pg/mL to 121.9 pg/mL and 101.7 pg/mL in the si-PD-1 and si-TGF-β groups, respectively. Compared with the si-PD-1 group, the level of TGF-β was greatly declined to 71.2 pg/mL in the si-PD-1 + si-TGF-β group (**p < 0.01 *vs*. si-NC, ##p < 0.01 *vs*. si-PD-1). In addition, the expression level of PD-1/PD-L1 was dramatically lower in the si-PD-1 and si-TGF-β groups and significantly lower in the si-PD-1 + si-TGF-β group than that in the si-NC group (*p < 0.05 *vs*. si-NC, **p < 0.01 *vs*. si-NC, ##p < 0.01 *vs*. si-PD-1).

### Proliferation and cytotoxicity of CD8^+^ T cells were synergistically enhanced by si-PD-1 combined with si-TGF-β

**Figure 3.**
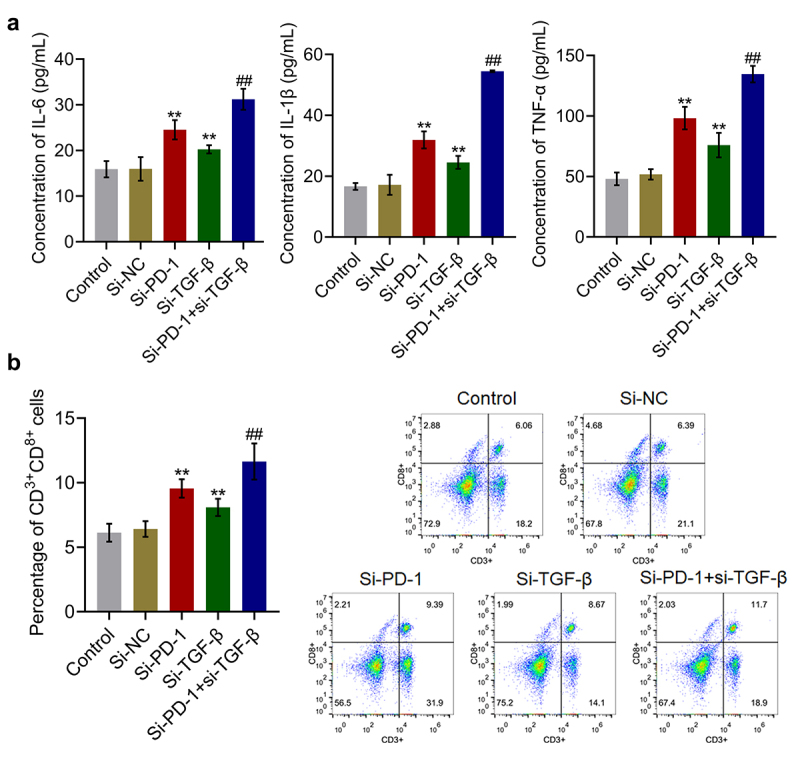
Si-PD-1 combined with si-TGF-β synergistically enhanced the proliferation and cytotoxicity of CD8^+^ T cells. RFA was performed on the co-culture of H22 and CD8^+^ T cells, followed by transfection with si-NC, si-PD-1, si-TGF-β, and si-PD-1 + si-TGF-β. A. The production of IL-6, IL-1β, and TNF-α was measured by ELISA. B. The percentage of CD3^+^CD8^+^ T cells was determined by flow cytometry (**p < 0.01 *vs*. si-NC, ##p < 0.01 *vs*. si-PD-1).

The levels of inflammatory factors and percentage of CD3^+^CD8^+^ T cells in the cell culture were measured to evaluate the cytotoxic effects of si-PD-1 and si-TGF-β on T cells. As shown in Figure 3a, compared with the si-NC group, the release of IL-6 was elevated from 15.9 pg/mL to 24.5 pg/mL and 20.3 pg/mL after transfection with si-PD-1 and si-TGF-β, respectively. Compared with the si-PD-1 group, the production of IL-6 was greatly promoted to 31.2 pg/mL in the si-PD-1 + si-TGF-β group. The concentrations of IL-1β in the control, si-NC, si-PD-1, si-TGF-β, and si-PD-1 + si-TGF-β groups were 16.7, 17.2, 31.9, 24.5, and 54.5 pg/mL, respectively. In addition, compared with the si-NC group, the production of TNF-α was elevated from 51.8 pg/mL to 98.2 pg/mL and 75.9 pg/mL after transfection with si-PD-1 and si-TGF-β, respectively. Further, compared with the si-PD-1 group, the secretion of TNF-α was greatly promoted to 134.6 pg/mL in the si-PD-1 + si-TGF-β group (**p < 0.01 *vs*. si-NC, ##p < 0.01 *vs*. si-PD-1). As shown in Figure 3b, compared with the si-NC group, the percentage of CD3^+^CD8^+^ T cells was elevated from 6.4% to 9.6% and 8.1% in the si-PD-1 and si-TGF-β groups, respectively, and was greatly increased to 11.6% after co-transfection with si-PD-1 and si-TGF-β.

### The PI3K/AKT/mTOR pathway was synergistically repressed by si-PD-1 combined with si-TGF-β

**Figure 4.**
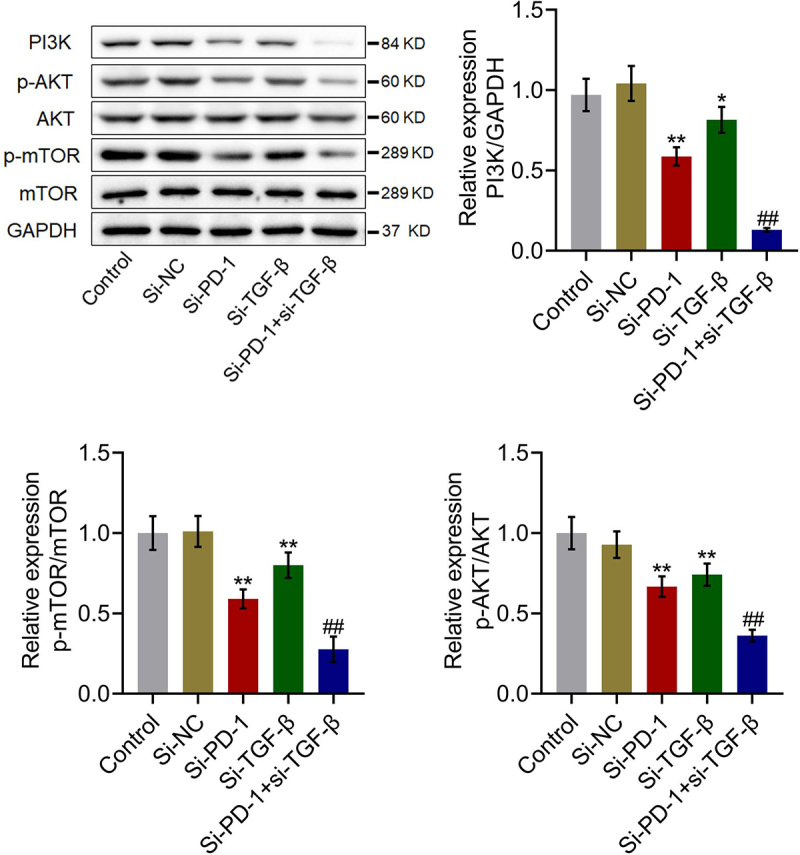
The PI3K/AKT/mTOR pathway was synergistically repressed by si-PD-1 combined with si-TGF-β. RFA was performed on the co-culture of H22 and CD8^+^ T cells, followed by transfection with si-NC, si-PD-1, si-TGF-β, and si-PD-1 + si-TGF-β. The expression levels of PI3K, AKT, p-AKT, mTOR, and p-mTOR was determined by western blotting (*p < 0.05 *vs*. si-NC, **p < 0.01 *vs*. si-NC, ##p < 0.01 *vs*. si-PD-1).

We further investigated the impact of si-PD-1 and si-TGF-β on the proliferation-related pathway in PLC cells. As shown in Figure 4, compared with si-NC, the expression levels of PI3K, p-AKT/AKT, and p-mTOR/mTOR were significantly downregulated by transfection with si-PD-1 and si-TGF-β. Compared with the si-PD-1 group, PI3K, p-AKT/AKT, and p-mTOR/mTOR were dramatically downregulated in the si-PD-1 + si-TGF-β group (*p < 0.05 *vs*. si-NC, **p < 0.01 *vs*. si-NC, ##p < 0.01 *vs*. si-PD-1). These data collectively revealed that the PI3K/AKT/mTOR pathway in PLC cells was synergistically inhibited by si-PD-1 combined with si-TGF-β.

### Tumor growth was suppressed and survival was prolonged synergistically by si-PD-1 combined with si-TGF-β

**Figure 5.**
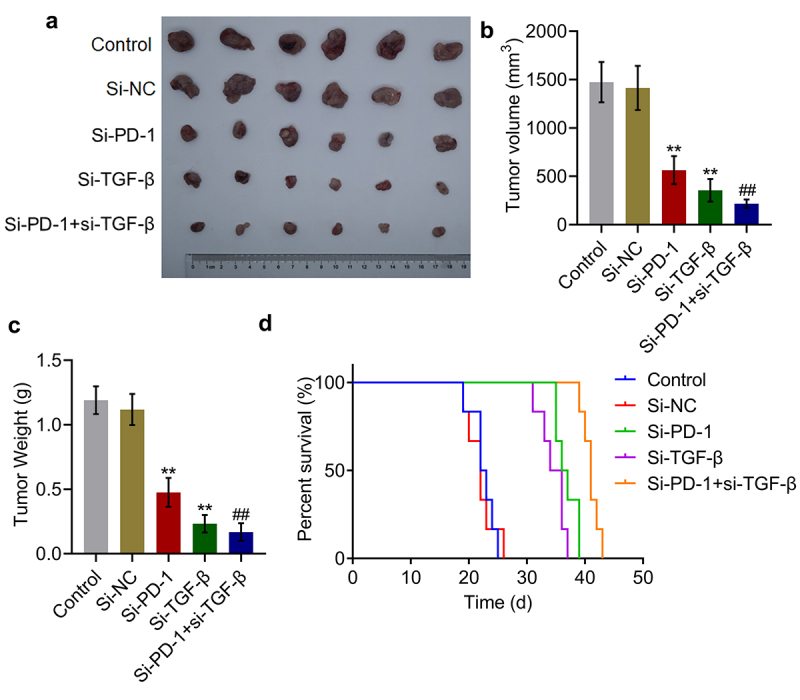
Si-PD-1 combined with si-TGF-β synergistically enhanced the inhibitory effect of RFA on tumor growth in H22 cell xenograft-bearing mice. H22 cells treated with blank medium, si-NC, si-PD-1, si-TGF-β, and si-PD-1 + si-TGF-β were transplanted into mice to establish the xenograft animal model; when the tumor size reached 100 mm^3^, RFA was performed. A. The image of tumor tissues. B. The volume of tumor tissues isolated from each group. C. The weight of tumor tissues isolated from each group. D. The percentage of survival as time went on in different group (**p < 0.01 *vs*. si-NC, ##p < 0.01 *vs*. si-PD-1).

To identify the synergistic antitumor effect of si-PD-1 combined with si-TGF-β, H22 cells treated with blank medium, si-NC, si-PD-1, si-TGF-β, and si-PD-1 + si-TGF-β were transplanted into mice to establish the xenograft animal model; when the tumor size reached 100 mm^3^, RFA was performed. As shown in Figure 5, compared with si-NC, significantly reduced tumor volume and weight, and prolonged survival were observed in the si-PD-1 and si-TGF-β groups. Compared with the si-PD-1 group, tumor volume and weight were dramatically declined accompanied by prolonged survival in the si-PD-1 + si-TGF-β group (**p < 0.01 *vs*. si-NC, ##p < 0.01 *vs*. si-PD-1). These data collectively indicated that si-PD-1 combined with si-TGF-β synergistically suppressed tumor growth and prolonged survival in RFA-treated H22 cell xenograft-bearing mice.

### TGF-β and PD-1/PD-L1 were suppressed by knockdown of PD-1 and TGF-β in tumor tissues

**Figure 6.**
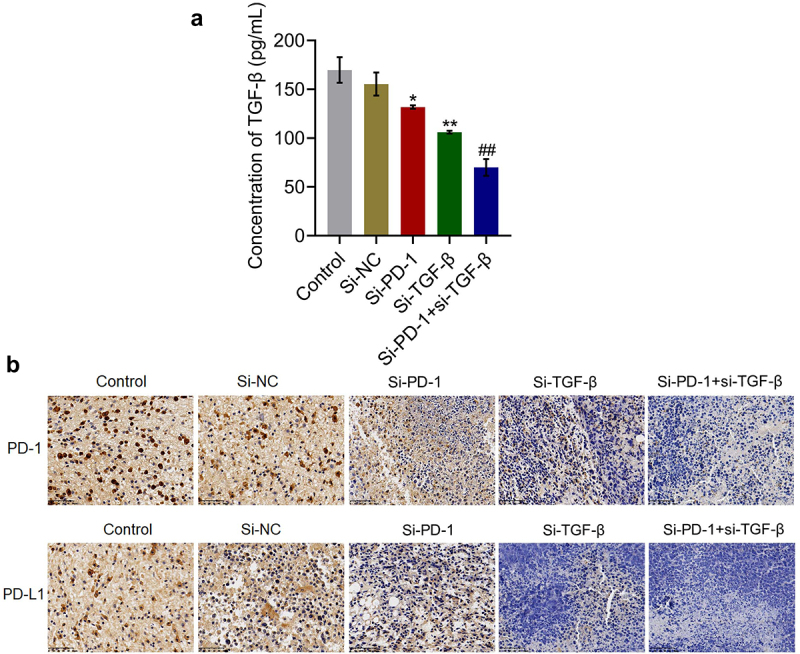
TGF-β and PD-1/PD-L1 were suppressed by si-PD-1 and si-TGF-β in tumor tissues. H22 cells treated with blank medium, si-NC, si-PD-1, si-TGF-β, and si-PD-1 + si-TGF-β were transplanted into mice to establish the xenograft animal model; when the tumor size reached 100 mm^3^, RFA was performed. A. The release of TGF-β in tumor tissues was measured by ELISA. B. The expression levels of PD-1 and PD-L1 were determined by immunohistochemical assay (*p < 0.05 *vs*. si-NC, **p < 0.01 *vs*. si-NC, ##p < 0.01 *vs*. si-PD-1).

We further verified whether the knockdown of PD-1 and TGF-β in tumor tissues was consistent with the results observed in *in vitro* studies. As shown in Figure 6a, compared with the si-NC group, the production of TGF-β in tumor tissues was significantly reduced from 155.4 pg/mL to 131.8 pg/mL and 106.1 pg/mL in the si-PD-1 and si-TGF-β groups, respectively. Compared with the si-PD-1 group, TGF-β secretion in tumor tissues was greatly declined to 70.0 pg/mL in the si-PD-1 + si-TGF-β group (*p < 0.05 *vs*. si-NC, **p < 0.01 *vs*. si-NC, ##p < 0.01 *vs*. si-PD-1). In addition, the expression levels of PD-1 and PD-L1 in the si-PD-1 and si-TGF-β groups were lower than those in the si-NC group, which was further downregulated in the si-PD-1 + si-TGF-β group.

### Percentage and cytotoxicity of CD8^+^ T cells were synergistically enhanced by si-PD-1 combined with si-TGF-β in tumor tissues

**Figure 7.**
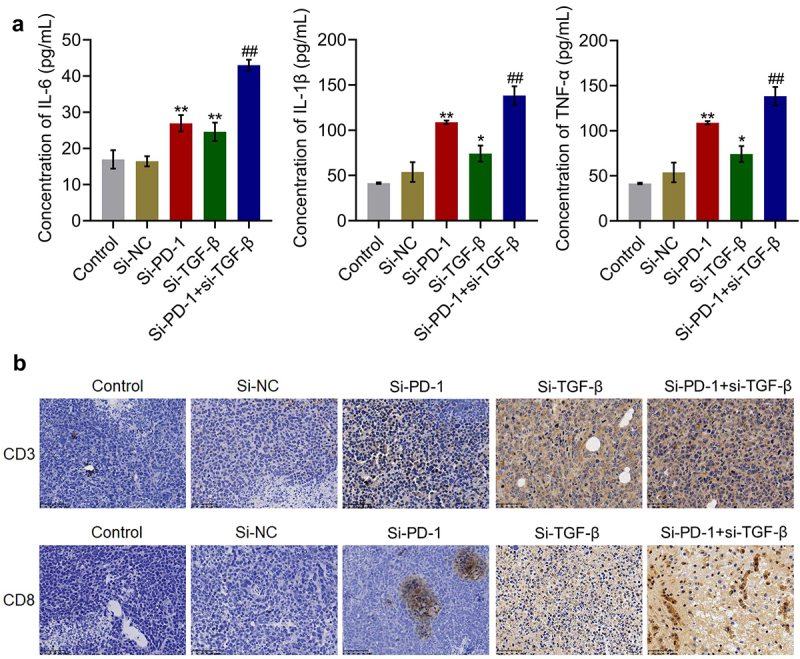
Si-PD-1 combined with si-TGF-β synergistically enhanced the percentage and cytotoxicity of CD8^+^ T cells in tumor tissues. H22 cells treated with blank medium, si-NC, si-PD-1, si-TGF-β, and si-PD-1 + si-TGF-β were transplanted into mice to establish the xenograft animal model; when the tumor size reached 100 mm^3^, RFA was performed. A. The production of IL-6, IL-1β, and TNF-α in tumor tissues was measured by ELISA. B. The percentage of CD3^+^CD8^+^ T cells in tumor tissues was determined by flow cytometry (*p < 0.05 *vs*. si-NC, **p < 0.01 *vs*. si-NC, ##p < 0.01 *vs*. si-PD-1).

The concentrations of inflammatory factors and expression levels of CD3 and CD8 in tumor tissues were measured. As shown in Figure 7a, compared with si-NC, the concentration of IL-6 was elevated from 16.5 pg/mL to 27.0 pg/mL and 24.6 pg/mL by transfection with si-PD-1 and si-TGF-β, respectively. Compared with the si-PD-1 group, the production of IL-6 was greatly promoted to 43.0 pg/mL in the si-PD-1 + si-TGF-β group. The concentrations of IL-1β in the control, si-NC, si-PD-1, si-TGF-β, and si-PD-1 + si-TGF-β groups were 18.2, 17.9, 31.9, 25.8, and 51.7 pg/mL, respectively. In addition, compared with si-NC, the production of TNF-α was elevated from 53.9 pg/mL to 109.0 pg/mL and 74.2 pg/mL by transfection with si-PD-1 and si-TGF-β, respectively. Compared with the si-PD-1 group, TNF-α secretion was significantly promoted to 138.5 pg/mL in the si-PD-1 + si-TGF-β group (*p < 0.05 *vs*. si-NC, **p < 0.01 *vs*. si-NC, ##p < 0.01 *vs*. si-PD-1). As shown in Figure 7b, compared with the si-NC group, the expression levels of CD3 and CD8 were elevated in the si-PD-1 and si-TGF-β groups. Compared with the si-PD-1 group, CD3 and CD8 were further upregulated in the si-PD-1 + si-TGF-β group.

### The PI3K/AKT/mTOR pathway in tumor tissues was synergistically inhibited by si-PD-1 combined with si-TGF-β

**Figure 8.**
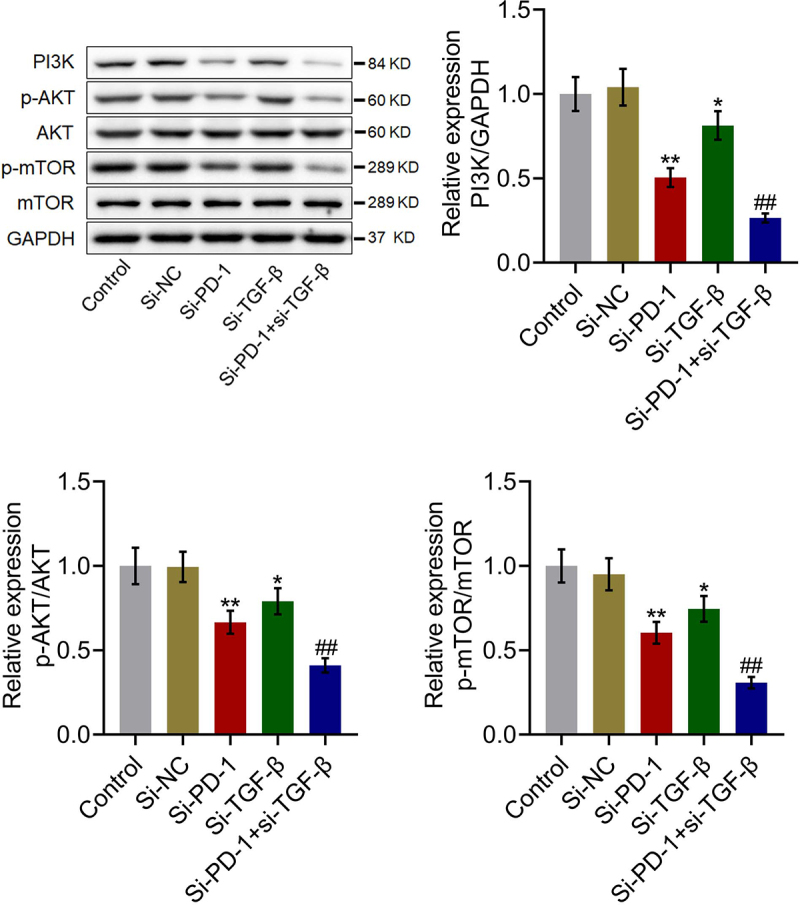
The PI3K/AKT/mTOR pathway in tumor tissues was synergistically inhibited by si-PD-1 combined with si-TGF-β. H22 cells treated with blank medium, si-NC, si-PD-1, si-TGF-β, and si-PD-1 + si-TGF-β were transplanted into mice to establish the xenograft animal model; when the tumor size reached 100 mm^3^, RFA was performed. The expression levels of PI3K, AKT, p-AKT, mTOR, and p-mTOR in tumor tissues were determined by western blotting (*p < 0.05 *vs*. si-NC, **p < 0.01 *vs*. si-NC, ##p < 0.01 *vs*. si-PD-1).

We further investigated the status of the PI3K/AKT/mTOR pathway in tumor tissues. As shown in Figure 8, compared with si-NC, PI3K, p-AKT/AKT, and p-mTOR/mTOR were significantly downregulated in the si-PD-1 and si-TGF-β groups. Compared with the si-PD-1 group, PI3K, p-AKT/AKT, and p-mTOR/mTOR were dramatically downregulated in the si-PD-1 + si-TGF-β group (*p < 0.05 *vs*. si-NC, **p < 0.01 *vs*. si-NC, ##p < 0.01 *vs*. si-PD-1). These data collectively revealed that the PI3K/AKT/mTOR pathway in tumor tissues of H22 cell xenograft-bearing animals was synergistically repressed by si-PD-1 combined with si-TGF-β.

## Discussion

Several studies have confirmed that RFA stimulates adaptive T-cell immunity in the tumor microenvironment, and exerts an inhibitory effect on tumors [21]. However, the antitumor effect of RFA is short-lived and insufficient to effectively prevent tumor recurrence. Moreover, RFA activates the immune response of T cells and induces adaptive immunosuppression, which is mainly manifested as inactivation of tumor-infiltrating T cells, amplification of Tregs and MDSCs in the tumor microenvironment, transformation of Th1 cells to Th2 cells, and induction of PD-L1 expression on the T cell surface after ablation [22]. At this point, the transformation of effector T cells to Treg cells after RFA treatment can be prevented by co-therapy with anti-PD-1 antibodies. Additionally, the expansion of MDSCs in tumors outside the ablation area can be inhibited by blocking PD-1 that significantly reduces the number of MDSCs that infiltrate tumor tissues, thus blocking and reversing immunosuppression. Maintaining antitumor immunity in the body after RFA treatment effectively reduces tumor recurrence and metastasis rates [23]. In the present study, we observed that compared with RFA monotherapy, co-treatment with RFA and si-PD-1 significantly repressed proliferation of H22 cells, facilitated apoptosis of H22 cells, considerably enhanced cytotoxicity of CD8^+^ T cells, and inhibited *in vivo* growth of H22 cells, indicating that a promising synergistic antitumor effect was achieved by combining RFA and blocking PD-1.

Studies have shown that RFA combined with PD-1 monoclonal antibody treatment results in more effective and prolonged antitumor immunity. However, the bottleneck that PD-1 monoclonal antibody benefits less than 20% of patient population with malignant tumor is still difficult to improve by the combination of RFA and PD-1 monoclonal antibody [24]. Therefore, exploring ways to improve the expression of PD-L1, thereby increasing the efficiency of PD-1/PD-L1 antibody therapy might be an effective method for the treatment of PLC. Studies have revealed that the regulatory effect of TGF-β on tumor growth is bidirectional. In the early stage, TGF-β functions as a tumor suppressor by inhibiting tumor growth. However, in the advanced stage, the TGF-β signaling pathway stimulates immune evasion of tumor cells by inhibiting the activation of T/B lymphocytes and natural killer cells, inhibiting the phagocytic ability of macrophages, and activating the classical Smad pathway or non-Smad pathway [25,26]. Moreover, proliferation of vascular endothelial cells can be facilitated by TGF-β, which is conducive to the formation of tumor tissues and plays a role in promoting tumor progression [27]. Several studies have shown that the occurrence and development of PLC are closely related to the activation of the TGF-β signaling pathway, and inhibition of this pathway represses the phenotypic differentiation of Tregs from Th1 to Th2, stimulates the role of Th1-type cytokines and M1-type macrophages, and activates the function of CD8^+^ T lymphocytes, natural killer cells, and dendritic cells. More importantly, PD-L1 is upregulated in tumor cells through targeted inhibition of TGF-β, which contributes to the improved efficacy of PD-1/PD-L1 therapy in PLC [28]. In the present study, under RFA treatment, we observed that compared with the PD-1 knockdown group, significantly decreased proliferation of H22 cells, increased apoptosis rate of H22 cells, enhanced cytotoxicity of CD8^+^ T cells, and decreased tumor growth in H22 cell xenograft model were observed in the PD-1 + TGF-β knockdown group, indicating that blocking TGF-β synergistically promoted the antitumor effect of RFA combined with PD-1 blockade therapy. Additionally, enhanced antitumor effect of the combination therapy with RFA, si-PD-1, and si-TGF-β was accompanied by the inhibition of the PI3K/AKT/mTOR pathway. In our future work, the crosstalk between the downstream of TGF-β and PI3K/AKT/mTOR pathways will be further investigated to support the potential application of combined RFA, PD-1 blocking, and TGF-β blocking therapy for the treatment of PLC.

The findings of the present study assure that the bottleneck of high metastasis and recurrence rates after PLC treatment with RFA can be solved by combining RFA with PD-1 blockade through suppression of the TGF-β signaling pathway. However, there are still some knowledge gaps such as species variation and individual differences. The future studies should verify synergistic antitumor effect through multicenter clinical trials to validate practical applications of the present findings. We believe that in the next five years, the combination of RFA with PD-1 blockade will be applied for the clinical treatment of PLC to benefit a large population of patients with PLC.

However, the present study has some limitations. First, only siRNA technology was used to block PD-1 and TGF-β to investigate the synergistic effect. However, in clinical treatment, inhibitors are widely used to block specific targets. Therefore, our future work will be focused on mutant PD-1 antibodies and TGF-β-specific inhibitors to further verify the synergistic effect of combination of RFA and PD-1 blockade for the treatment of PLC.

## Conclusion

Our data revealed that blocking the TGF-β signaling pathway produced a synergistic effect of combination therapy of PD-1 blockade and RFA against PLC.

## References

[1] Kudo M. Surveillance, diagnosis, treatment, and outcome of liver cancer in Japan. Liver Cancer. 2015;4:39–50. 26020028 10.1159/000367727 PMC4439792

[2] Rizzo A, Brandi G. Biochemical predictors of response to immune checkpoint inhibitors in unresectable hepatocellular carcinoma. Cancer Treat Res Commun. 2021;27:100328. 33549983 10.1016/j.ctarc.2021.100328

[3] Rizzo A, Ricci AD, Brandi G. Immune-based combinations for advanced hepatocellular carcinoma: shaping the direction of first-line therapy. Future Oncol. 2021;17:755–757. 33508960 10.2217/fon-2020-0986

[4] Rizzo A, Dadduzio V, Ricci AD, et al. Lenvatinib plus pembrolizumab: the next frontier for the treatment of hepatocellular carcinoma? Expert Opin Investig Drugs. 2021;1–8. 10.1080/13543784.2021.1948532 34167433

[5] Sun T, Samiotaki G, Wang S, et al. Acoustic cavitation-based monitoring of the reversibility and permeability of ultrasound-induced blood-brain barrier opening. Phys Med Biol. 2015;60:9079–9094. 26562661 10.1088/0031-9155/60/23/9079 PMC4668271

[6] Izzo F, Granata V, Grassi R, et al. Radiofrequency ablation and microwave ablation in liver tumors: an update. Oncologist. 2019;24:e990–e1005. 31217342 10.1634/theoncologist.2018-0337 PMC6795153

[7] Li D, Kang J, Madoff DC. Locally ablative therapies for primary and metastatic liver cancer. Expert Rev Anticancer Ther. 2014;14:931–945. 24746315 10.1586/14737140.2014.911091

[8] Nishimura M, Nouso K, Kariyama K, et al. Safety and efficacy of radiofrequency ablation with artificial ascites for hepatocellular carcinoma. Acta Med Okayama. 2012;66:279–284. 22729109 10.18926/AMO/48568

[9] Wen L, Xin B, Wu P, et al. An efficient combination immunotherapy for primary liver cancer by harmonized activation of innate and adaptive immunity in mice. Hepatology. 2019;69:2518–2532. 30693544 10.1002/hep.30528 PMC6541536

[10] Fujita M, Yamaguchi R, Hasegawa T, et al. Classification of primary liver cancer with immunosuppression mechanisms and correlation with genomic alterations. EBioMedicine. 2020;53:102659. 32113157 10.1016/j.ebiom.2020.102659 PMC7048625

[11] Kudo M. Immune checkpoint inhibition in hepatocellular carcinoma: basics and ongoing clinical trials. Oncology. 2017;92(Suppl 1):50–62. 28147363 10.1159/000451016

[12] Philips GK, Atkins M. Therapeutic uses of anti-PD-1 and anti-PD-L1 antibodies. Int Immunol. 2015;27:39–46. 25323844 10.1093/intimm/dxu095

[13] Qi X, Li G, Liu D, et al. Development of a radiofrequency ablation platform in a clinically relevant murine model of hepatocellular cancer. Cancer Biol Ther. 2015;16:1812–1819. 26537481 10.1080/15384047.2015.1095412 PMC4847992

[14] Montoya JJ, Azorsa DO. Optimization of transfection conditions for siRNA screening. Methods Mol Biol. 2016;1470:15–24. 27581281 10.1007/978-1-4939-6337-9_2

[15] Kumar P, Nagarajan A, Uchil PD. Analysis of cell viability by the MTT assay. Cold Spring Harb Protoc. 2018;2018(6). 10.1101/pdb.prot095505 29858338

[16] Jurisic V, Srdic-Rajic T, Konjevic G, et al. TNF-alpha induced apoptosis is accompanied with rapid CD30 and slower CD45 shedding from K-562 cells. J Membr Biol. 2011;239:115–122. 21221555 10.1007/s00232-010-9309-7

[17] Zhong YB, Zhang XL, Lv MY, et al. Detection of IL-1beta, IL-6 and TNF-alpha in Sprague-Dawely rats’ atrophic thymus induced by lipopolysaccharide. Pol J Vet Sci. 2018;21:589–597. 30468342 10.24425/124294

[18] Jurisic V. Multiomic analysis of cytokines in immuno-oncology. Expert Rev Proteomics. 2020;17:663–674. 33131355 10.1080/14789450.2020.1845654

[19] Song Y, Wang JG, Li RF, et al. Gecko crude peptides induce apoptosis in human liver carcinoma cells in vitro and exert antitumor activity in a mouse ascites H22 xenograft model. J Biomed Biotechnol. 2012;2012:743573. 23093861 10.1155/2012/743573 PMC3471029

[20] Vieth M, Kushima R, Mukaisho K, et al. Immunohistochemical analysis of pyloric gland adenomas using a series of Mucin 2, Mucin 5AC, Mucin 6, CD10, Ki67 and p53. Virchows Arch. 2010;457:529–536. 20827489 10.1007/s00428-010-0968-7

[21] Chen DS, Mellman I. Oncology meets immunology: the cancer-immunity cycle. Immunity. 2013;39:1–10. 23890059 10.1016/j.immuni.2013.07.012

[22] Duffy AG, Ulahannan SV, Makorova-Rusher O, et al. Tremelimumab in combination with ablation in patients with advanced hepatocellular carcinoma. J Hepatol. 2017;66:545–551. 27816492 10.1016/j.jhep.2016.10.029 PMC5316490

[23] El-Khoueiry AB, Sangro B, Yau T, et al. Nivolumab in patients with advanced hepatocellular carcinoma (CheckMate 040): an open-label, non-comparative, phase 1/2 dose escalation and expansion trial. Lancet. 2017;389:2492–2502. 28434648 10.1016/S0140-6736(17)31046-2 PMC7539326

[24] Shi L, Wang J, Ding N, et al. Inflammation induced by incomplete radiofrequency ablation accelerates tumor progression and hinders PD-1 immunotherapy. Nat Commun. 2019;10:5421. 31780645 10.1038/s41467-019-13204-3 PMC6883042

[25] Colak S, Ten Dijke P. Targeting TGF-beta signaling in cancer. Trends Cancer. 2017;3:56–71. 28718426 10.1016/j.trecan.2016.11.008

[26] Itatani Y, Kawada K, Sakai Y. Transforming growth factor-beta signaling pathway in colorectal cancer and its tumor microenvironment. Int J Mol Sci. 2019; 20(23):5822. 10.3390/ijms20235822 PMC6929101 31756952

[27] Tauriello DVF, Palomo-Ponce S, Stork D, et al. TGFbeta drives immune evasion in genetically reconstituted colon cancer metastasis. Nature. 2018;554:538–543. 29443964 10.1038/nature25492

[28] Mariathasan S, Turley SJ, Nickles D, et al. TGFbeta attenuates tumour response to PD-L1 blockade by contributing to exclusion of T cells. Nature. 2018;554:544–548. 29443960 10.1038/nature25501 PMC6028240

